# Identification of a novel variant in N-cadherin associated with dilated cardiomyopathy

**DOI:** 10.3389/fmed.2022.944950

**Published:** 2022-08-30

**Authors:** Yuanying Chen, Qiqing Sun, Chanjuan Hao, Ruolan Guo, Chentong Wang, Weili Yang, Yaodong Zhang, Fangjie Wang, Wei Li, Jun Guo

**Affiliations:** ^1^Beijing Key Laboratory for Genetics of Birth Defects, Beijing Pediatric Research Institute, MOE Key Laboratory of Major Diseases in Children, Capital Medical University, Center of Rare Diseases, National Center for Children's Health, Beijing Children's Hospital, Capital Medical University, Beijing, China; ^2^Henan Key Laboratory of Pediatric Inherited and Metabolic Diseases, Children's Hospital Affiliated to Zhengzhou University, Zhengzhou Hospital of Beijing Children's Hospital, Zhengzhou, China; ^3^Department of Cardiology, Children's Hospital Affiliated to Zhengzhou University, Zhengzhou Hospital of Beijing Children's Hospital, Zhengzhou, China

**Keywords:** N-cadherin, dilated cardiomyopathy, cell-cell adhesion, *de novo* variant, whole exome sequencing

## Abstract

**Background:**

Dilated cardiomyopathy (DCM), which is a major cause of heart failure, is a primary cardiac muscle disease with high morbidity and mortality rates. DCM is a genetically heritable disease and more than 10 gene ontologies have been implicated in DCM. *CDH2* encodes N-cadherin and belongs to a superfamily of transmembrane proteins that mediate cell–cell adhesion in a calcium-dependent manner. Deficiency of *CDH2* is associated with arrhythmogenic right ventricular cardiomyopathy (OMIM: 618920) and agenesis of the corpus callosum, cardiac, ocular, and genital syndrome (OMIM: 618929). However, there have been no reports of isolated DCM associated with *CDH2* deficiency.

**Methods:**

We performed whole exome sequencing in a 12-year-old girl with non-syndromic DCM and her unaffected parents. Variants in both known DCM-related genes and novel candidate genes were analyzed and pathogenicity confirmation experiments were performed.

**Results:**

No pathogenic/likely pathogenic variant in known DCM-related genes was identified in the patient. We found a *de novo* variant in a candidate gene *CDH2* in the patient, namely, c.474G>C/p.Lys158Asn (NM_001792.5). This variant has not been reported in the ClinVar or Human Gene Mutation Database (HGMD). CDH2 p.Lys158Asn was found in the conserved domain of N-cadherin, which is associated with the hydrolysis of the precursor segment and interference with adhesiveness. Furthermore, we tested the expression and efficiency of cell–cell adhesion while overexpressing the CDH2 Lys158Asn mutant and two previously reported variants in *CDH2* as positive controls. The adhesion efficiency was considerably reduced in the presence of the mutated CDH2 protein compared with wild-type CDH2 protein, which suggested that the mutated CDH2 protein's adhesion capacity was impaired. The variant was probably pathogenic after integrating clinical manifestations, genetic analysis, and functional tests.

**Conclusion:**

We identified a *CDH2* variant in DCM. We observed a new clinical symptom associated with N-cadherin deficiency and broadened the genetic spectra of DCM.

## Introduction

Dilated cardiomyopathy (DCM), which is a major cause of heart disorders, is a primary cardiac muscle disease with high morbidity and mortality rates (1). The estimated incidence and prevalence of DCM is 1:2,500–3,000 (2). However, DCM might be much more common, and the frequency could be 1 in 250 individuals (3). The genetic basis of DCM is highly complex and diverse. Gene mutations contributing to DCM affect the function of proteins, including sarcomere, cytoskeleton, nuclear envelope, γ-secretase activity, ion channel, mitochondrial, spliceosomal, sarcoplasmic reticulum, and desmosomal proteins (3). There are more than 50 DCM-associated genes, but only 12 genes (*BAG3, DES, DSP, FLNC, LMNA, MYH7, PLN, RBM20, SCN5A, TNNC1, TNNT2*, and *TTN*) are classified as having a definitive or strong relationship with DCM (4, 5).

The *CDH2* gene encodes N-cadherin, which is a classical type I cadherin that plays a critical role in cell–cell adhesion in the nervous system and the heart (6–8). Rare heterozygous *CDH2* variants are associated with the occurrence of human diseases. Accogli et al. reported nine individuals with variants in *CDH2* who showed agenesis of the corpus callosum, cardiac, ocular, and genital (ACOG) syndrome (9). Variants of *CDH2* also cause arrhythmogenic right ventricular cardiomyopathy (ARVC) (10–12), Peters anomaly (13), and brain arteriovenous malformation (14) in a few cases. Besides the biological relevance of N-cadherin in human illnesses, animals lacking N-cadherin in certain tissues show impaired internal cortex structures (15) and cardiac function (16–18).

In this study, we identified a *de novo* heterozygous *CDH2* variant, c.474G>C/p.Lys158Asn (NM_001792.5), in a non-syndromic patient who had DCM without other abnormalities using trio whole exome sequencing. To explain the pathogenicity, we ectopically overexpressed wild-type CDH2 protein and its variants in HeLa cells. We found that the adhesion efficiency was considerably lower when carrying the mutated CDH2 proteins than that with the wild-type protein, which indicated that the adhesive ability of mutated N-cadherin was impaired. To the best of our knowledge, there have been no reports of *CDH2* deficiency in isolated DCM. We identified a novel *CDH2* variant and extended the genotype–phenotype spectrum of DCM.

## Materials and methods

### Participants

The proband was a girl aged 12 years who was admitted to the Department of Cardiology, Zhengzhou Hospital of Beijing Children's Hospital because of edema in both lower limbs without obvious inducement. This study was approved by the Institutional Review Board (IRB) of Beijing Children's Hospital, Capital Medical University (Ethics Approval Number 2015-26). Informed consent was obtained from the subject and her parents. The use of patient-specific information and images was granted by her parents.

### Whole exome sequencing, bioinformatics analysis, and Sanger sequencing

**Figure 1:**
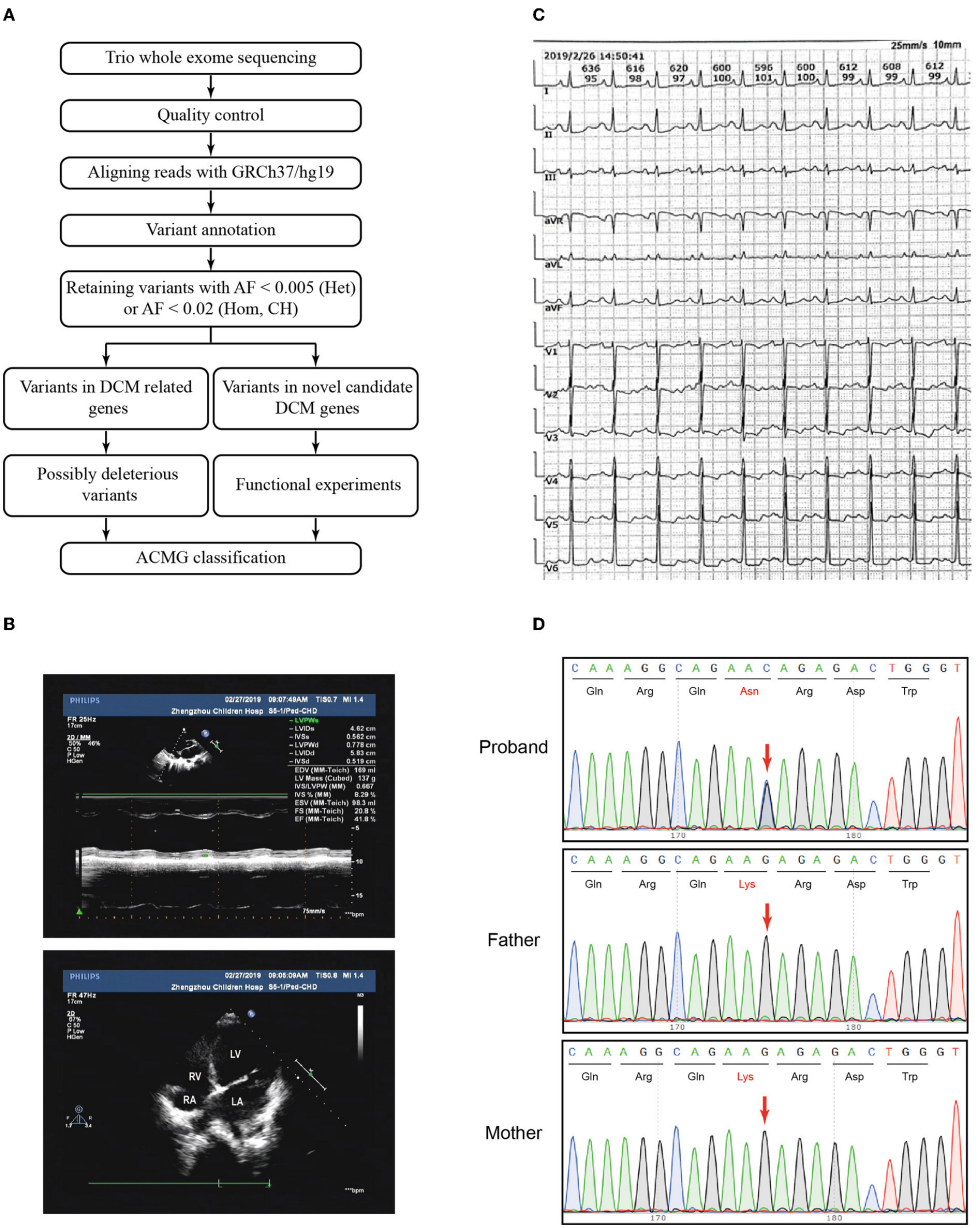
Variant filtration workflow and the phenotype of the patient. **(A)** Variant filtration workflow employed in this study. AF: allele frequency; Het, heterozygous; Hom, homozygous; CH, compound heterozygous; DCM, dilated cardiomyopathy; ACMG, American College of Medical Genetics and Genomics. **(B)** The echocardiography showed an apical four-chamber view of the proband's indicating dilation of the right ventricle. RV, right ventricle; RA, right atrium; LV, left ventricle; LA, left atrium. **(C)** Electrocardiogram (ECG) showed sinus rhythm, P wave changes, and ST-T changes. **(D)** Sanger sequencing confirmed that the variant *CDH2* c.474G>C was heterozygous in the proband and not inherited from her parents. The arrows indicated the mutated nucleotides.

Genomic DNA of peripheral blood was extracted, purified, and broken into random segments. The genomic DNA was then captured using the Agilent SureSelect Human All Exome V6 Kit (Agilent Technologies, Santa Clara, CA, USA), and a sequencing library was prepared. Using the Illumina Hiseq X Ten sequencer (Illumina, San Diego, CA, USA), we carried out whole exome sequencing with a reading length of 150 bp. The data obtained from sequencing (raw data) were subject to quality control, basic data analysis, and filtering to remove joint sequences and repeat sequences. Sequence alignment was performed according to the GRCh37/hg19 human reference genome sequence, and the single-nucleotide polymorphisms and insertion–deletion polymorphisms of samples were annotated. Sequencing depth, coverage, and homogeneity were counted. Variants were filtered according to the analytical workflow (Figure 1A). We analyzed the variant frequency and filtered out the common variants with an allele population variation >0.5%, or >2% if the variant was homozygous or if there was a second variant in the gene using 1000 Genome, dbSNP, gnomAD, and in-house databases. Variants were classified into two categories: variants in DCM-related genes and variants in novel candidate DCM genes. Fifty-one genes curated by a DCM Gene Curation Expert Panel that proposed to have a monogenic role in isolated, idiopathic DCM in humans were considered as DCM-related genes (Supplementary Table S1) (5). Prediction software, such as Polyphen-2 (http://genetics.bwh.harvard.edu/pph2/), SIFT (http://sift.jcvi.org), Mutation Taster (http://www.mutationtaster.org), and CADD (http://cadd.gs. washington.edu/), was used to evaluate the harmfulness of the variants. Loss-of-function (LoF) variants, such as stop gain, stop loss, frameshift indels, and splice site variants (2 nt plus/minus the exon boundary), as well as missense variants predicted to be deleterious by at least two prediction tools were defined as possibly deleterious variants. The pathogenicity of variants was classified in accordance with the American College of Medical Genetics and Genomics (ACMG) Standards and Guidelines (19). Sanger sequencing was performed using the ABI 3730xl DNA Analyzer (Applied Biosystems, MA, USA). The used oligonucleotides are chr18-25591882-Forward 5'-GGCTTTCTACAACACTACAGAAAT-3' and chr18-25591882-Reverse 5'-ACTGTGATTCCTATGCTTTCAGGT-3'.

### Constructs and site-directed mutagenesis

The *CDH2* cDNA library for the human N-cadherin cloning template was purchased from OriGene (China). Using the site-directed mutagenesis strategy, wild-type CDH2 (pCMV6-CDH2-Flag) and mutation constructs (pCMV6-CDH2 Lys158Asn-Flag, pCMV6-CDH2 Asp407Asn-Flag, and pCMV6-CDH2 Asp597Asn-Flag) were successfully established using KOD-Plus Neo (TOYOBO, Japan) and Dpn I (Thermo Fisher Scientific, USA). The primers used to generate amplicons of *CDH2*-specific variation sites are shown in Supplementary Table S2. Positive clones were verified by sequencing.

### Western blot

HeLa cells (Zhejiang Meisen Cell Technology Co., Ltd. Catalog number: CTCC-001-0006) were transfected, collected, and lysed using cell lysis buffer (Beyotime Biotechnology, China). Sodium dodecyl sulfate-polyacrylamide gel electrophoresis (SDS-PAGE) was performed using a 4–15% gradient gel (Applygen Technologies, China). Proteins were electrophoretically transferred onto a polyvinylidene fluoride (PVDF) membrane and subjected to immunoblotting. Flag and β-actin antibodies were purchased from Sigma (Merck, Germany) with a dilution of 1:1000 in Western blot.

### Cell–cell adhesion assay

The cell–cell adhesion assay was conducted following the protocol as described by Accogli et al. with slight modifications (9). HeLa cells were transfected with a pCMV6-Flag vector (Control), pCMV6-CDH2-Flag (CDH2), pCMV6-CDH2 Lys158Asn-Flag (CDH2 Lys158Asn), pCMV6-CDH2 Asp407Asn-Flag (CDH2 Asp407Asn), and pCMV6-CDH2 Asp597Asn-Flag (CDH2 Asp597Asn) constructs for 24 h. Transfected cells were trypsinized and seeded in triplicate (1 × 10^5^/well) into a 24-well plate with extracellular buffer (140 mM NaCl, 5 mM KCl, 10 mM glucose, and 10 mM HEPES) containing 2 mM Ca^2+^ and 1% bovine serum albumin. The cells were then incubated for 1 h at 37°C for aggregate formation. An aggregate was defined as >4 cells. The aggregated cell number (A1) and the total cell number (A0) were counted. The fraction of cell adhesion was defined as A1/A0.

### Statistical analysis

Data are shown as mean ± standard deviation of three independent experiments. One-way analysis of variance was used to conduct statistical analysis, and Bonferroni's correction was used to perform multiple comparisons. The significance value was set at *p* < 0.05.

## Results

### Clinical description

The proband had intermittent edema of both lower limbs for 14 days. She was born full-term with a birth weight of 3.15 kg and had normal physical and mental development. While growing up, she had poor physical condition. She usually had difficulty in movement, easily sweated after activities, easily vomited, and had abdominal discomfort. According to her history, the patient appeared to have chronic cardiac insufficiency. The patient's parents were non-consanguineous and reported no family history of cardiomyopathy.

### Echocardiography and diagnosis

The patient and her parents had echocardiography performed. The patient showed an enlarged left atrium and left ventricle, mild to moderate mitral regurgitation, mild tricuspid regurgitation, and slightly decreased left ventricular systolic function (Figure 1B, Table 1). Electrocardiogram (ECG) showed sinus rhythm, P wave changes, and ST-T changes (Figure 1C). Combined with her previous medical history, a diagnosis of DCM was considered (1). Echocardiography showed that the left ventricular end diastolic dimension (LVDd) of the patient was 58.3 mm, which is much larger (>117%) than normal left ventricular diastolic dysfunction values. The patient's left ventricular ejection fraction was 42%, and fractional shortening (FS) was 21%, which met the diagnostic criteria of DCM. The patient's parents had no symptoms of heart failure, and their echocardiographic findings were normal. The proband and her parents were enrolled in this study. Blood samples were obtained to conduct whole exome sequencing because gene mutations that contribute to DCM are highly diverse and complex.

**Table 1:** Measurement data of echocardiography.

**Name**	**Measurement**	**Name**	**Measurement**	**Name**	**Measurement**
LAD	51.6 mm	RAD	34.3 mm	RVOT	20.1 mm
MPA	21.5 mm	Vmax	0.59 m/s	LPA	9.4 mm
RPA	10.1 mm	AoD	18.1 mm	Vmax	0.84 m/s
MVE	0.79 m/s	MVA	0.47 m/s	TVE	0.49 m/s
TVA	0.31 m/s	RVDd	13.9 mm	IVSTd	5.2 mm
LVDd	58.3 mm	LVPWd	6.5 mm	LVDs	46.2 mm
EDV	169 ml	ESV	98.3 ml	SV	70.7 ml
EF	42%	FS	21%	DAO	0.98 m/s

LAD, left atrium dimension; MPA, main pulmonary artery; RPA, right pulmonary artery; MVE, mitral valve inflow E wave velocity; TVA, tricuspid valve inflow A wave velocity; LVDd, left ventricular end diastolic dimension; EDV, end diastolic volume; EF, ejection fraction; RAD, right atrium dimension; Vmax, maximum velocity; AoD, ascending aorta dimension; MVA, mitral valve inflow A wave velocity; RVDd, right ventricular end diastolic dimension; LVPWd, left ventricular posterior wall dimension; ESV, end-systolic volume; FS, fractional shortening; RVOT, right ventricular outflow tract; LPA, left pulmonary artery; TVE, tricuspid valve inflow E wave velocity; IVSTd, interventricular septum time dimension; LVDs, left ventricular end-systolic dimension; SV, stroke volume; DAO, descending aorta.

### Genetic analysis

Trio whole exome sequencing was performed using peripheral blood DNA from the patient with DCM and her unaffected parents. A total of 354 rare variants in the proband (Supplementary Table S3) were detected. Five rare heterozygous variants in DCM-related genes were identified and 3 of which were possibly deleterious variants in this patient (Supplementary Table S4), including a variant in *MYH6* inherited from the patient's father and 2 variants in *TTN* inherited from the patient's father and mother, respectively. No pathogenic/likely pathogenic variant in DCM-related genes was identified in the proband according to the ACMG guidelines. To identify variants in novel candidate DCM genes, we evaluated the homozygous, compound heterozygous, and *de novo* variants after variant filtration, and found a *de novo CDH2* variant, NC_000018.9(NM_001792.5):c.474G>C, NP_001783.2:p.(Lys158Asn), in the patient. Subsequent Sanger sequencing confirmed that the variant was heterozygous in the proband and not inherited from her parents (Figure 1D).

**Figure 2:**
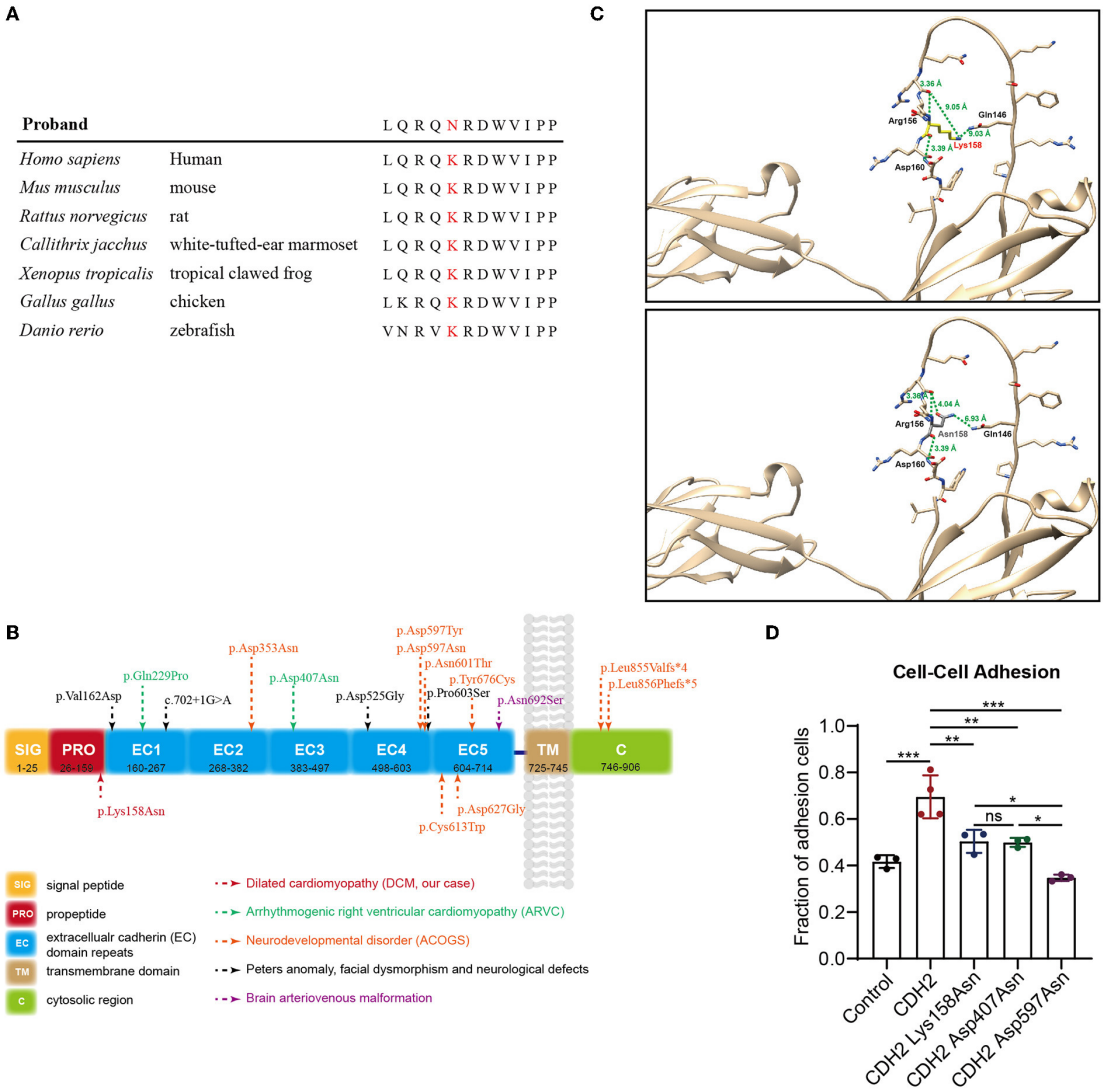
Genetic analysis and functional tests of the *de novo* variant (c.474G>C/p.Lys158Asn) in *CDH2*. **(A)** Sequence alignment of human CDH2 and other species shows the conservation of the affected residue (p.Lys158Asn). **(B)** Schematic representation of the topological domains of CDH2 protein. Rare heterozygous *CDH2* variants linked to ARVC, ACOGs, Peters anomaly, and brain arteriovenous malformation were annotated. **(C)** The structure of CDH2 and Lys158Asn mutant. The human CDH2 protein structure was modeled from the “AlphaFold Protein Structure Database” (AlphaFoldDB: P19022, https://alphafold.com/). The structure was aligned with the solution structure of neural cadherin prodomain (PDB: 1OP4) and analyzed using UCSF chimera. **(D)** The cell-cell adhesion efficiency of the wild-type and CDH2 variations. The significance value was set as *p* < 0.05 (*), *p* < 0.01 (**), and *p* < 0.001 (***).

This variant has not been reported in the Human Gene Mutation Database (HGMD) or ClinVar database and is not present in population databases. Multiple *in silico* prediction tools supported a pathogenic effect (Table 2) of this variant. Sequence alignment indicated that the variant was conserved among most species (Figure 2A). CDH2 is structurally divided into a signal peptide, a cadherin propeptide, five extracellular cadherin repeats, a transmembrane region, and a cytoplasmic tail. Recently, several rare heterozygous *CDH2* variants encoding extracellular cadherin repeats and the cytosolic region were associated with ARVC, ACOG syndrome, Peters anomaly, and arteriovenous malformation in the brain (Figure 2B). The CDH2 Lys158Asn variant in our proband is located at the propeptide domain, which is responsible for endoproteolytic cleavage. Amino acid mutations in the propeptide (Arg-X_1_-Lys-Arg-X_2_-Trp, i.e., R-X_1_-K-R-X_2_-W polypeptide) lead to the inability of hydrolysis of the precursor segment and might interfere with adhesiveness.

**Table 2:** Population Allele frequency and predicted pathogenicity score of the *CDH2* variant.

	***NC_000018.9(NM_001792.5):c.4***
	***74G>C, NP_001783.2:p.(Lys158Asn)***
**Gene**	*CDH2*
**Chromosome location**	chr18:25591882
**Transcript**	NM_001792.5
**Amino acid change**	p.Lys158Asn
**Zygosity**	Heterozygous
**Allele frequency in population**	
gnomAD_exome East Asian	-
1000 genomes	-
dbSNP	-
ExAc	-
***In silico*** **prediction**	
SIFT	Damaging (score: 0.002)
Polyphen-2_HVAR	Probably damaging (score: 0.998)
MutationTaster	Disease causing (score: 1.0)
CADD	Damaging (score: 33)

### Functional experiments

In the first place, we analyze the structure of CDH2 and Lys158Asn mutant. The human CDH2 protein structure was modeled from the “AlphaFold Protein Structure Database” (AlphaFoldDB: P19022, https://alphafold.com/). The structure was aligned with the solution structure of neural cadherin prodomain (PDB: 1OP4) and analyzed using UCSF chimera (20). The CDH2 Lys158Asn mutant was located in a long unstructured loop. We evaluated the hydrogen bond forming, the distances between amino acids, the structural clashes, and the molecule contacts. We found that the backbone of the 158 lysine (Lys158) residue can form polar interactions between nearby 156 Arginine (Arg156) and 160 aspartic acid (Asp160). The lysine residue changed into asparagine (Asn158) did not affect backbone polar interaction. Whereas the side chain has a violent change of conformation, thus the remote distances (9.05 Å and 9.03 Å) from 146 glutamine (Gln146) changed into 4.04 Å and 6.93 Å and may impair the endoproteolytic cleavage (Figure 2C). Then, functional experiments were performed to further evaluate the pathogenicity of the *CDH2* variant. We cloned *CDH2* and its variants into a flag-tagged pCMV6-Entry vector using site-directed mutagenesis. Two additional *CDH2* variants, p.Asp407Asn and p.Asp597Asn, were used as positive controls, because they have been proved to be pathogenic or experimentally demonstrate to impair cell–cell adhesion. An empty flag-tagged pCMV6-Entry vector was used as a negative control. Control plasmids, the wild-type, and constructs carrying *CDH2* variations were transfected into HeLa cells and seeded for Western blot and cell–cell adhesion assays. There were no significant differences in protein concentrations between the wild-type and CDH2 Lys158Asn and CDH2 Asp407Asn. The protein expression level of CDH2 Asp597Asn was 40% higher than that of the wild-type (Supplementary Figures S1A,B), which may be an artifact of overexpression. We then normalized protein expression levels and conducted a cell–cell adhesion assay. The cell–cell adhesion efficiency of CDH2 Lys158Asn was approximately 30% lower compared with the wild-type CDH2 (Figure 2D). Additionally, the cell–cell adhesion efficiency of CDH2 Asp597Asn was 20% lower than that of CDH2 Lys158Asn and CDH2 Asp407Asn. This finding indicated a relationship between the loss of mediating the cell–cell adhesion ability and pathogenicity. According to the ACMG guidelines, the *CDH2* variant, NC_000018.9(NM_001792.5):c.474G>C and NP_001783.2:p.(Lys158Asn), was classified as pathogenic (PS2+PS3+PM2+PP3) after integrating genetic analysis and functional tests.

## Discussion

In this study, no pathogenic/likely pathogenic variant in 51 DCM-related genes based on previous reports (Supplementary Table S1) was identified in the proband. However, we identified a *de novo CDH2* heterozygous missense variant, c.474G>C/p.Lys158Asn.

Classical cadherin proteins are involved in the cell–cell adhesion process (21, 22). The classical cadherins are subdivided into types I and II and are comprised of five repeated extracellular (EC1–5) regions. The extracellular domain of cadherins provides three calcium-binding pockets and interacts with other cadherins in *cis* and *trans*. The cytoplasmic tail of cadherins binds with adaptor proteins, such as catenins, which connect cadherins to the actomyosin cytoskeleton (21, 23). *CDH2* encodes N-cadherin, which is a transmembrane protein and a critical calcium-dependent factor that mediates cell–cell adhesion (24). N-cadherin is highly expressed in the heart and other tissues, such as neurons. In heart tissues, N-cadherin is expressed in a structure called the intercalated disc, through which cardiomyocytes are extensively interconnected (25). The intercalated disc ensures tight electromechanical coupling of cardiomyocytes, thus protecting the integrity and function of the myocardium. This highly organized structure comprises three junction complexes of gap junctions, desmosomes, and adherens junctions. The gap junction is essential for intercellular communications, especially rapid electrical transmission between cells (26). The desmosomes provide structural support *via* interactions of desmosomal cadherins with the filament system desmin (27). Adherens junctions are composed of a classic cadherin in the heart, N-cadherin, and mediate calcium-dependent cell adhesion (28). The junction complexes of the intercalated disc must be organized properly to maintain the normal function of myocytes. The two desmosomal cadherins desmocollin-2 (*DSC2*) and desmoglein-2 (*DSG2*) mediate cell–cell adhesion of cardiomyocytes. Heterozygous or homozygous variants of *DSC2* and *DSG2* have been described in patients with arrhythmogenic cardiomyopathy (29–31).

Limited data have implicated the involvement of N-cadherin in the pathogenesis of arrhythmogenic cardiomyopathy in humans. A previous study identified a *CDH2* missense variant (c.686A>C, p.Gln229Pro) in a three-generation family with ARVC by whole exome sequencing. Subsequently, the authors examined 73 ARVC probands with negative mutations in known ARVC genes and found another likely pathogenic variant in *CDH2* (c.1219G>A, p.Asp407Asn) (10). Another study performed whole exome sequencing and genomic triangulation in a pedigree, as well as in a 14-year-old female proband with arrhythmogenic cardiomyopathy, her affected mother and sister, and her unaffected father. The authors found that *CDH2* c.1219G>A (p.Asp407Asn) was likely a pathogenic variant and concluded that *CDH2* was a novel autosomal dominant susceptibility gene for arrhythmogenic cardiomyopathy (11). Reevaluation cohort of genetic variants associated with ARVC confirmed these *CDH2* variants (12). Recently, *CDH2* variants were reported to be associated with a Mendelian neural developmental disorder. Nine individuals with a syndromic neural developmental disorder were characterized by ACOG syndrome (9). This study also showed that the variants in *CDH2* impaired the adhesive activity of N-cadherin. Therefore, *CDH2* appears to cause human genetic diseases, such as ARVC and ACOG syndrome.

Several animal models have been used to determine the essential role of N-cadherin in the structural integrity of the heart and the relationship between N-cadherin and cardiac development and function. Ferreira-Cornwell et al. established a transgenic mouse model using the mouse myosin heavy chain (MHC) promoter, which can specifically express chicken N-cadherin in the heart (αMHC/Ncad). They found that the size of αMHC/Ncad hearts was larger than that in non-transgenic mice. Histological analysis showed a dilated left ventricle and thinner ventricular wall in αMHC/Ncad hearts, which suggested that the modulation of cadherin-mediated adhesion contributed to DCM (32). Another study generated N-cadherin conditional knockout mice using a cardiac-specific tamoxifen-inducible Cre transgene, which resulted in N-cadherin deletion in the myocardium. The authors from this previous study showed that intercalated discs, adherens junctions, and desmosomes disappeared. The mutant mice displayed moderate DCM and died because of arrhythmia (16). Another similar mouse model showed that the expression of the gap junction proteins connexin-43 and connexin-40 was significantly decreased in N-cadherin cardiac-restricted knockout mice, which led to a ventricular conduction defect (17). *CDH2* mutant zebrafish also showed an enlarged pericardial cavity and disorganized atrium and ventricle, which suggests a vital role of N-cadherin in cardiac development in zebrafish (18). Studies in animal models have suggested that N-cadherin produces the phenotype of DCM whereby it may be one of the pathogenic genes of DCM.

In our study, the patient has a large left ventricle without arrhythmia. Typical ARVC, balanced ARVC, and right ventricular dominant ARVC were not considered. There was no low voltage of QRS wave complex in limb lead and T-wave inversion in inferior wall lead in ECG, which did not support a phenotype of left ventricular dominant ARVC. So, we considered a diagnosis of idiopathic DCM. Then, we found the *de novo CDH2* variant c.474G>C (p.Lys158Asn) in the patient. This variant has not been reported in the ClinVar or HGMD. Sanger sequencing confirmed that the missense variant was heterozygous in the proband and absent in her unaffected parents. There were no facial or neurodevelopmental abnormalities in the proband. During hospitalization, digoxin, hydrochlorothiazide, spironolactone, aspirin tablets, and potassium citrate granules were used for the treatment. The proband improved and was discharged from the hospital. A follow-up study was conducted for 1 year. Initially, the proband's condition was stable. The proband's edema then became more serious, and she was hospitalized in Beijing Children's Hospital and the local hospital three times intermittently, where maintenance treatment was carried out. The edema and arrhythmia of the proband became aggravated. Her left ventricular ejection fraction was 33% at the last hospitalization. An electrocardiogram showed frequent ventricular premature beats. The patient then developed arrhythmia and died at age 13. All of the phenotypes were consistent with the manifestation of DCM.

Subsequent studies showed that the *CDH2* variant p.Lys158Asn was located in the N-cadherin prodomain and was conserved in most species. The adhesive capacity of cadherins relies on the removal of the prodomain because cleavage of the precursor peptide is required for the maturation of N-cadherin. The endogenous cleavage site mutation may not affect the N-cadherin targeting the plasma membrane but may disturb the interaction between N-cadherins to form homodimers (33, 34). Previous studies have shown that endogenous protease digestion depends on the recognition site (Arg-X_1_-Lys-Arg-X_2_-Trp, i.e., R-X_1_-K-R-X_2_-W polypeptide) (35). After the original recognition sites changed to specific digestion sites of trypsin and factor Xa, they cannot be recognized by the endogenous protease. Mutations in the endogenous recognition sites interfere with adhesive activities. Therefore, the N-cadherin mutation p.Lys158Asn in endogenous protease digestion sites may affect the proteolysis of the prodomain and subsequently affect the function of N-cadherin (35, 36). Our experiment using transfected HeLa cells with the *CDH2* Lys158Asn mutant showed unchanged protein expression levels and lower adhesion efficiency compared with those in the wild-type. This finding suggests that the N-cadherin prodomain also plays a vital role in mediating cell adhesion. Moreover, the differences in adhesion efficiency in HeLa cells between CDH2 Asp597Asn and CDH2 Lys158Asn may explain the various phenotypes. In conclusion, we identified a novel *CDH2* variant, c.474G>C (p.Lys158Asn), in a patient with nonsyndromic DCM by exome sequencing. We discovered a new clinical phenotype of this *CDH2* variant and extended the currently known genetic variety of DCM.

## Data availability statement

The original contributions presented in the study are included in the article/Supplementary material, further inquiries can be directed to the corresponding author/s. Whole exome sequencing data were not deposited because of the low number of patients described in this manuscript, to protect patient privacy and confidentiality.

## Ethics statement

The studies involving human participants were reviewed and approved by the Ethics Committee of Beijing Children's Hospital, Capital Medical University. Written informed consent to participate in this study was provided by the participants' legal guardian/next of kin. Written informed consent was obtained from the individual(s), and minor(s)' legal guardian/next of kin, for the publication of any potentially identifiable images or data included in this article.

## Author contributions

YC and JG designed and performed most of the experiments, analyzed the data, and wrote the manuscript. QS collected clinical data of the proband. CH and RG helped to validate the data. CW and WY helped with plasmids construction. YZ, FW, and WL helped to organize the clinical data. JG were responsible for forming the hypothesis, project development, data coordination, writing, finalizing, and submitting the manuscript. All authors have read and approved the manuscript.

## Funding

This research was supported by the Ministry of Science and Technology of China (2016YFC1000306), the National Natural Science Foundation of China (81770234 and 31830054), the Beijing Municipal Science and Technology Commission Foundation (Z181100001918003), and the Beijing Municipal Commission of Health and Family Planning Foundation (ShouFa 2018-2-1141).

## Conflict of interest

The authors declare that the research was conducted in the absence of any commercial or financial relationships that could be construed as a potential conflict of interest.

## Publisher's note

All claims expressed in this article are solely those of the authors and do not necessarily represent those of their affiliated organizations, or those of the publisher, the editors and the reviewers. Any product that may be evaluated in this article, or claim that may be made by its manufacturer, is not guaranteed or endorsed by the publisher.
