# Supplementary material for: Identification of a novel variant in N-cadherin associated with dilated cardiomyopathy
Source: Front Med (Lausanne). 2022 Aug 30;9:944950. doi: 10.3389/fmed.2022.944950 (PMC9468813; doi:10.3389/fmed.2022.944950)
Supplement: Supplementary file 1 [file Table_1.DOCX]

| *BAG3* | *DSP* | *CSRP3* | *MYH6* | *TBX20* |
| --- | --- | --- | --- | --- |
| *DES* | *ACTC1* | *CTF1* | *MYL2* | *TCAP* |
| *FLNC* | *ACTN2* | *DSG2* | *MYPN* | *TNNI3K* |
| *LMNA* | *JPH2* | *DTNA* | *NEBL* | *LRRC10* |
| *MYH7* | *NEXN* | *EYA4* | *NKX2-5* | *NPPA* |
| *PLN* | *TNNI3* | *GATAD1* | *OBSCN* | *MIB1* |
| *RBM20* | *TPM1* | *ILK* | *PLEKHM2* | *MYL3* |
| *SCN5A* | *VCL* | *LAMA4* | *PRDM16* | *PDLIM3* |
| *TNNC1* | *ABCC9* | *LDB3* | *PSEN2* | *PKP2* |
| *TNNT2* | *ANKRD1* | *MYBPC3* | *SGCD* | *PSEN1* |
| *TTN* |  |  |  |  |

**Supplemental Table 1:** A set of 51 genes resulting in proposed to have a monogenic role in isolated, idiopathic DCM in humans (1).

Reference

1 Jordan, E., Peterson, L., Ai, T., Asatryan, B., Bronicki, L., Brown, E., et al. Evidence-Based Assessment of Genes in Dilated Cardiomyopathy. *Circulation*. (2021) 144:7-19. doi: 10.1161/CIRCULATIONAHA.120.053033
