# Supplementary material for: Identification of a novel variant in N-cadherin associated with dilated cardiomyopathy
Source: Front Med (Lausanne). 2022 Aug 30;9:944950. doi: 10.3389/fmed.2022.944950 (PMC9468813; doi:10.3389/fmed.2022.944950)
Supplement: Supplementary file 2 [file Table_2.DOCX]

**Supplementary Table 2**: Primers used to generate *CDH2* variations

| Primers | 5’-3’ |
| --- | --- |
| *CDH2* Lys158Asn Forward | CCTACAAAGGCAGAA**C**AGAGACTGGGTCATC |
| *CDH2* Lys158Asn Reverse | GATGACCCAGTCTCT**G**TTCTGCCTTTGTAGG |
| *CDH2* Asp407Asn Forward | AATCTAACTGTGACC**A**ATAAGGATCAACCCC |
| *CDH2* Asp407Asn Reverse | GGGGTTGATCCTTAT**T**GGTCACAGTTAGATT |
| *CDH2* Asp597Asn Forward | CAGATCTATTTACTT**A**ATATTAATGACAATG |
| *CDH2* Asp597Asn Reverse | CATTGTCATTAATAT**T**AAGTAAATAGATCTG |
