# Supplementary material for: Identification of a novel variant in N-cadherin associated with dilated cardiomyopathy
Source: Front Med (Lausanne). 2022 Aug 30;9:944950. doi: 10.3389/fmed.2022.944950 (PMC9468813; doi:10.3389/fmed.2022.944950)
Supplement: Supplementary file 4 [file Table_4.DOCX]

**Supplementary Table 4:** Rare variants in DCM-related genes in the patient

| Gene | Omim Inheritance | Transcript | cDNA | Protein | Zygosity | | | SIFT | PP2 | MT | CADD | ACMG |
| --- | --- | --- | --- | --- | --- | --- | --- | --- | --- | --- | --- | --- |
|  |  |  |  |  | Patient | Father | Mother |  |  |  |  |  |
| ***MYH6*** | **AD** | **NM_002471.4** | **c.224A>G** | **p.Gln75Arg** | **HET** | **HET** | **-** | **D** | **B** | **D** | **23.0** | **VUS** |
| ***TTN*** | **AD;AR** | **NM_003319.4** | **c.68831T>G** | **p.Ile22944Arg** | **HET** | **HET** | **-** | **D** | **B** | **D** | **21.8** | **VUS** |
| *TTN* | AD;AR | NM_003319.4 | c.29170G>C | p.Val9724Leu | HET | - | HET | T | - | N | 15.94 | VUS |
| *TTN* | AD;AR | NM_133378.4 | c.30583_30597delACAGAGAAGAAAGTG | p.Thr10195_Val10199del | HET | HET | - | - | - | - | - | VUS |
| ***TTN*** | **AD;AR** | **NM_133378.4** | **c.22124A>T** | **p.Asp7375Val** | **HET** | **-** | **HET** | **T** | **B** | **D** | **22.8** | **VUS** |

AD, autosomal dominant; AR, autosomal recessive; HET, heterozygous; HEM, hemizygous; SIFT (D: damaging; T: tolerate); PP2, Polyphen-2_HVAR (B: Benign); MT, Mutation Taster (D: disease causing, N: polymorphism); CADD, Combined Annotation Dependent Depletion; ACMG, American College of Medical Genetics and Genomics guidelines (VUS, variants of uncertain significance); -, not found; possibly deleterious missense or LoF (Loss-of-Function) variants are indicated in bold.
