# Supplementary figures and images for: Identification of a novel variant in N-cadherin associated with dilated cardiomyopathy
Source: Front Med (Lausanne). 2022 Aug 30;9:944950. doi: 10.3389/fmed.2022.944950 (PMC9468813; doi:10.3389/fmed.2022.944950)

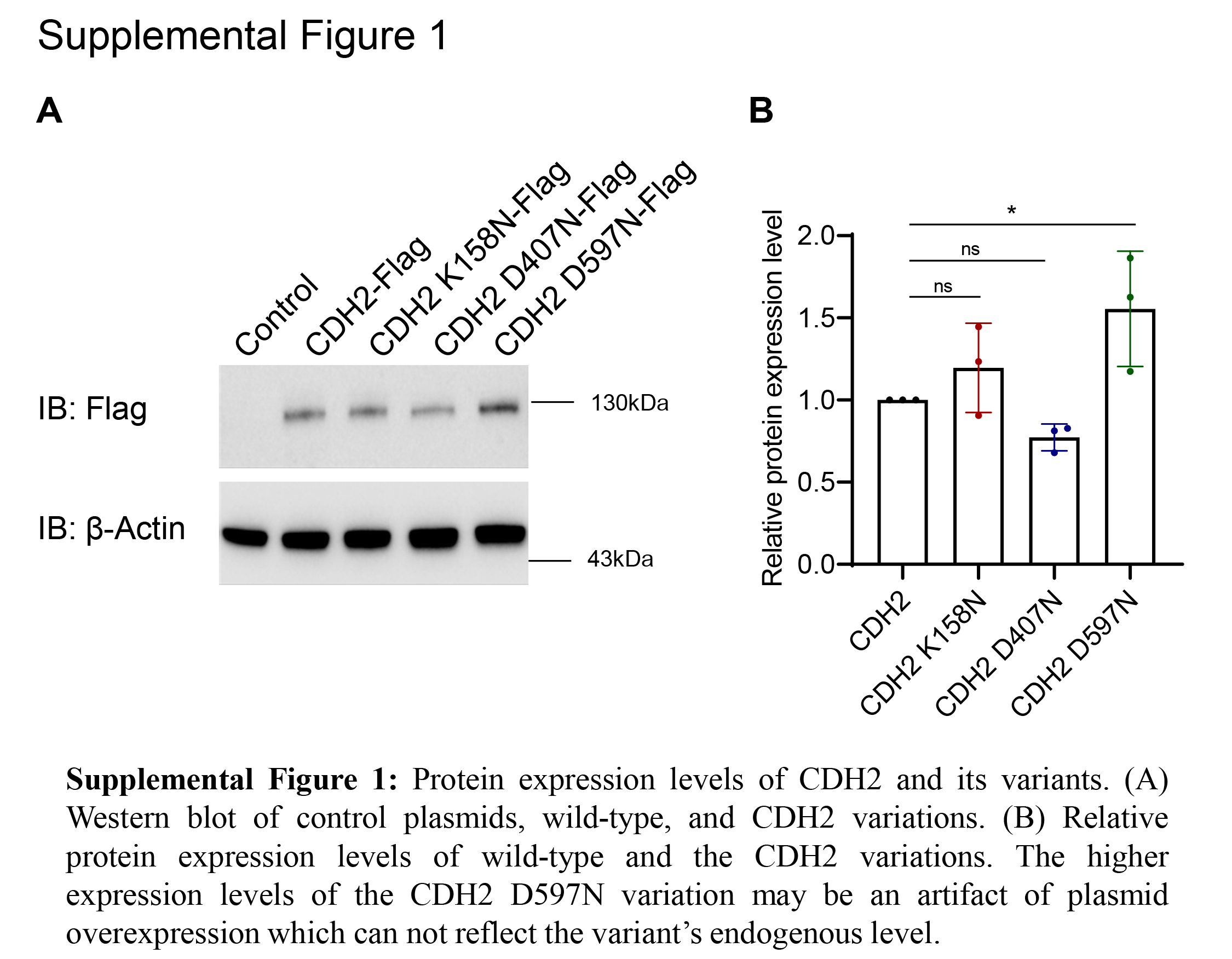

Supplement: Supplementary file 5 [file Image_1.TIF]
